# Cost-Effectiveness of Eplerenone Compared to Usual Care in Patients With Chronic Heart Failure and NYHA Class II Symptoms, an Australian Perspective

**DOI:** 10.1097/MD.0000000000003531

**Published:** 2016-05-06

**Authors:** Zanfina Ademi, Kumar Pasupathi, Danny Liew

**Affiliations:** From the Department of Epidemiology and Preventive Medicine (DEPM), Monash University, Melbourne, Australia (ZA, DL), Institute of Pharmaceutical Medicine, University of Basel, Basel, Switzerland (ZA), and Optum (KP), Sydney, Australia.

## Abstract

The objective of this study was to determine the cost-effectiveness of eplerenone compared with usual care in patients with chronic heart failure and New York Heart Association (NYHA) Class II symptoms.

A Markov model was constructed with 5 health states to reflect NYHA symptom status (Classes I–IV) and death. All subjects began in the “Class II” health state and then moved to other symptom health states or died. Subjects could also be hospitalized for HF in any cycle. Transition probabilities were derived from the Eplerenone in Mild Patients Hospitalization And Survival Study in Heart Failure (EMPHASIS-HF) study. Decision analysis was applied to compare an Eplerenone Group with a Usual Care Group (UCG). In the UCG, 47.3% of subjects in Class II and 93.7% of subjects in Classes III and IV were assumed to be taking spironolactone (as per published data). In the Eplerenone Group, all subjects in Classes II, III, and IV were assumed to be taking eplerenone. The efficacy of spironolactone was assumed to be the same as eplerenone. Cost and utility data were derived from published sources. A discount rate of 5.0% was applied to future costs and benefits. The outcome of interest was incremental cost-effectiveness ratio (ICER) (cost per year of live saved (YoLS) and quality-adjusted life years (QALY) gained).

Over 10 years the model predicted that for each patient compared with usual care, eplerenone would lead to 0.26 YoLS (discounted) and 0.19 QALYs gained (discounted), at a net cost of AUD $6961 (discounted). These equate to ICERs of AUD 28,001 per YoLS and AUD 37,452 per QALY gained. Sensitivity analyses indicated a 99.0% likelihood of eplerenone being cost-effective compared with usual care at a willingness to pay threshold of AUD 50,000 per QALY gained.

From an Australian healthcare perspective, the addition of eplerenone in management of patients with chronic heart failure and NYHA Class II symptoms represents a cost-effective strategy compared with usual care.

## Abstract 2

Supplemental Digital Content is available in the text

## INTRODUCTION

Chronic heart failure (CHF) imposes a great burden of morbidity and mortality in the world.^1–4^ Current estimates of the prevalence of CHF range from 1.0% to 2.0%.^2,3,5^ In Australia, epidemiological data about the prevalence of CHF are scarce, but the estimated incidence is 5 to 10 per 1000 individuals, per year.^6^ Associated healthcare costs are high, with at least AUD 1 billion dollars of healthcare devoted annually to CHF, which is of similar magnitude to that of stroke.^5^ Notably, the burden of CHF in Australia, like many other Western countries, is expected to increase due to an aging population and better survival from acute cardiac diseases.^7^

Guideline recommendations for the management of patients with CHF and New York Heart Association (NYHA) Class II symptoms include angiotensin-converting enzyme inhibitors, angiotensin II receptor blockers, and beta-blockers, with the key aim of relieving symptoms and prolong survival.^4,5^ In addition, based on recent evidence from the Eplerenone in Mild Patients Hospitalization and Survival Study in Heart Failure (EMPHASIS-HF) study,^8^ the aldosterone receptor antagonist eplerenone should also be considered. In EMPHASIS-HF, 2737 patients with NYHA Class II heart failure and an ejection fraction of not more than 35% were randomized to take either eplerenone (up to 50 mg daily) or a placebo, in addition to recommended therapy. The primary outcome was a composite of death from cardiovascular causes or hospitalization for heart failure. The study was stopped prematurely after a median follow-up of 21 months. The primary outcome occurred in 18.3% and 25.9% of the eplerenone and placebo groups, respectively, equating to a hazard ratio (HR) of 0.63 (95% confidence interval [CI], 0.54–0.74). Overall, mortality (HR 0.76, 95% CI 0.62–0.93; *P* = 0.008) and cardiovascular mortality (HR 0.76, 95% CI, 0.61–0.94) were also reduced by using eplerenone.

Recently, we undertook a modelled cost-effectiveness analysis of eplerenone compared with placebo, among patients initially with NYHA Class II CHF, based on the perspective of the Australian healthcare system.^9^ However, there were 2 main limitations to our analysis. First, our modeled analysis did not explicitly consider the progression of patients from NYHA Class II symptoms to Class III and IV symptoms. Instead, model subjects were simply simulated to experience hospitalization for heart failure or die. Second, we assumed that there was no use of spironolactone among model subjects. These 2 assumptions are overly simplistic because in current practice some patients with NYHA Class II symptoms would be taking spironolactone, and the number would increase as they progressed to more severe symptom stages.

Hence, the aim of the present analysis was to assess the cost-effectiveness of eplerenone compared with usual care, which included use spironolactone, among patients initially with NYHA Class II CHF.

## METHODS

**FIGURE 1:**
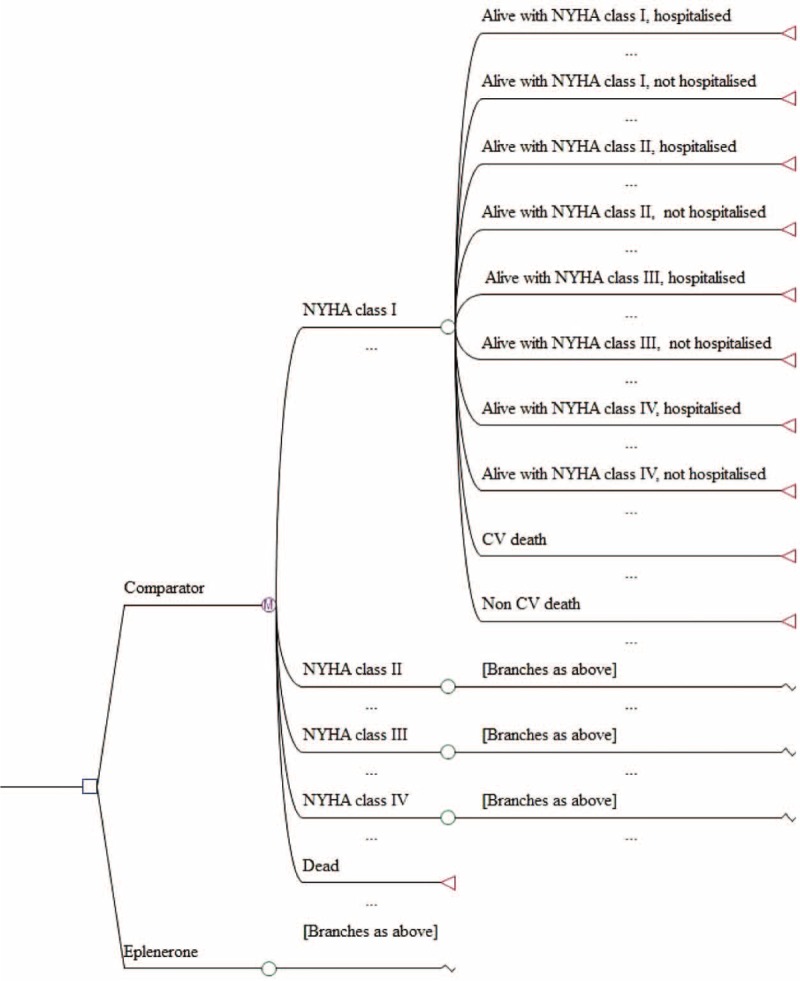
Decision analytic in combination with Markov model. Model input data for transition probabilities.

We implemented a state transition Markov model^10^ with 1 year cycles to reflect the status of subjects with initial NYHA Class II CHF, and their progression to other NYHA classes over a 10-year time horizon. A Markov model is the most common modeling technique used to simulate the long-term health and economic outcomes of condition.^11,12^ Decision tree analysis^13^ was applied to compare downstream morbidity, mortality, and costs incurred by an “Eplerenone group” (EG) and a “Usual Care Group” (UCG). The model consisted of 5 health states: “Alive, with NYHA I,” “Alive, with NYHA II,” “Alive, with NYHA III,” “Alive, with NYHA IV,” and “Dead” (Figure 1). The simulated model started in the health state “Alive, with NYHA II” and progressed through 10 transition probabilities, which were similar for all living health states (Figure 1). Below is the list of transition probabilities that participants underwent through the model.

“NYHA Class I no hospitalization, stay alive”; “NYHA Class I hospitalization, stay alive” (hospitalization comprised of an overnight stay or longer in a hospital environment with a discharge diagnosis that included a cardiovascular reason); “NYHA Class II no hospitalization, stay alive”; “NYHA Class II hospitalization, stay alive”; “NYHA Class III, no hospitalization, stay alive”; “NYHA Class III hospitalization, stay alive”; “NYHA Class IV, no hospitalization, stay alive”; “NYHA Class IV hospitalization, stay alive”; “cardiovascular death” (included heart failure, myocardial infarction, cardiac arrhythmia, stroke, or other cardiovascular causes) regardless of what other nonfatal events (including hospitalization) may have occurred in that cycle prior to death; and “noncardiovascular death” despite other nonfatal events potentially having occurred in that cycle prior to death.

Hospitalization was defined as incidence of any number of heart failure hospitalizations (≥1) and survival until the end of the cycle. Any deaths taking place during the cycle were assumed to be mutually exclusive to nonfatal heart failure hospitalizations.

The health economic evaluation model was designed from an Australian healthcare system perspective. The time horizon of the model was 10 years, reflecting the timeline of other published sources, and the model's baseline used 2014 Australian dollars (AUD). The main outcome of interest was number needed to treat and cost-effectiveness of eplerenone versus UCG was expressed as incremental cost-effectiveness ratios (ICER) in terms of AUD per years of life saved (YoLS) and AUD per quality adjusted life year (QALY) gained. As this is secondary research, ethical approval is not required.

### Model Population

The present analysis was based on the results from the EMPHASIS-HF trial,^8^ a randomized double-blinded trial, which was conducted in 29 countries around the world. Baseline characteristics of chronic HF patients recruited in Australia were comparable to overall patients recruited in the global EMPHASIS trial. Therefore, clinical findings are transferable to the Australian population. In Appendix 1, baseline demographics of EMPHASIS patients are described. The main inclusion criteria for the EMPHASIS trial ranged from patients having symptoms of NYHA Class II CHF, age ≥55, and a Left Ventricular Ejection Fraction (LVEF) of ≤30% (unless LVEF is >30% and ≤35%, on an electrocardiogram (ECG), where the QRS duration must be >130 msec). Patients must also be on ACE inhibitor and/or angiotensin receptor blocker treatment and beta-blocker treatment (unless contra-indicated).^8^

The model design allows for progression of patients from NYHA Class II symptoms to Class III and IV symptoms, in both the EG and UCG. The UCG consisted of a proportion of subjects taking either spironolactone or placebo treatment.

In the base case analyses, an assumption was made that 43.7% of patients with NYHA Class II symptoms will be on spironolactone treatment and 93.7% of patients with NYHA Class III–IV symptoms respectively. This information was derived from responding physicians in the Parnicka et al study,^14^ which was a nationwide educational project on HF management in primary care in Poland. Based on this study, 43.7%^14^ of physicians prescribed spironolactone among patients with NYHA Class II symptoms. 93.7%^14^ of physicians prescribed spironolactone among patients with NYHA Class III–IV symptoms. There is no study available comparing the effect of spironolactone treatment in NYHA Class II to placebo or even to eplerenone. Therefore, the proportion of patients in the UCG using spironolactone in NYHA Class II and NYHA Class III and IV respectively (43.7%, 93.7%) will have the efficacy of eplerenone (described below in the section of transition probabilities for eplerenone).

The remaining proportion of subjects in the UCG were then assigned to placebo. Therefore, the percentage of patients in the UCG assigned to placebo was 100%, 56.3%, and 6.3% for patients with NYHA Class I, Class II, and Classes III–IV respectively.

The transition probabilities among patients within each NYHA Class in the UCG using placebo were derived from the EMPHASIS clinical trial. The EMPHASIS clinical trial provided individual data over 4 years of follow-up within each NYHA Class in the UCG using placebo. In the base-case analysis, we have used weighted average values for all cycles (10-year time horizon, for the UCG using placebo). However, in the scenario analyses that follow, we have used individual patient data over 4 years of follow-up for the UCG using placebo, followed by weighted average values for cycle 5 and beyond to understand the effect on the main outcomes.

Table 1 describes transition probabilities of subjects in the UCG on placebo, their related hospitalizations, and deaths within each NYHA Class (Table 1). In addition, another assumption was made that transition probabilities for NYHA Class IV were the same as the NYHA Class III transition probabilities derived from the EMPHASIS trial on placebo arm (Table 1). Furthermore, the Australian Institute of Health and Welfare's (AIHW) General Record of Incidence of Mortality^15^ (Appendix 2) was used to derive age-related trends that allowed for extrapolation of transition probabilities from Cycles 1 to 2 and beyond. This process allows cardiovascular and noncardiovascular death risks derived from EMPHASIS to incorporate age-related trends. The mean age at baseline in the EMPHASIS trial was 68 years; therefore, this was the assumed age of patients in Cycle 1 of the model.

**TABLE 1:**
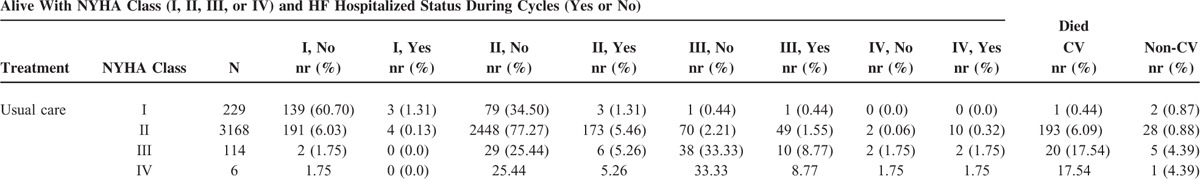
Transition Probabilities for Usual Care Arm, Among Subjects With New York Heart Association Class (I, II, III, or IV)

Transition probabilities for eplerenone group were obtained by multiplying the hazard ratios (HRs) for “eplerenone versus placebo” to the weighted average transition probabilities from the placebo arm in the EMPHASIS trial (Table 1).^8^ For heart failure hospitalizations the HR was 0.58 (95% CI: 0.47–0.70), all-cause mortality had an HR of 0.76 (95% CI: 0.62–0.93), and cardiovascular death had an HR of 0.76 (95% CI: 0.61–0.94).^8^ In the base-case analyses the assumption was made that HRs at baseline remain constant throughout the modeled time horizon.

### Utilities

Since no utility data exists showing differences between eplerenone and placebo or spironolactone stratified by NYHA Class, the utility values used in the model were derived from a study that assessed the impact of medical therapy, alone or with cardiac resynchronization, among patients with NYHA Class III and IV, with an average age of 66 years.^16^ These utilities were also reported in the long-term cost-effectiveness of cardiac resynchronization therapy, with or without an implantable cardioverter-defibrillator by Yao et al.^17^ Furthermore, these values have been used in other heart failure cost-effectiveness studies,^9,18^ and are the only heart failure utility values that stratify by NYHA Class. These were estimated from quality-of-life assessments made during CARE-HF, using the EQ-5D at baseline and 90 days.^16^ As such, both the usual treatment and eplerenone groups of the model used these utility values. NYHA Class I had a utility of 0.815, NYHA Class II a utility of 0.720, NYHA Class III a utility of 0.590, and NYHA Class IV a utility of 0.508. No utility decrement was applied to hospitalizations.

### Costs (Australian Dollars)

A weighted average of the relevant 14th Round of Australian Refined Diagnosis Related Groups (AR-DRGs) (2009–2010) was used to cost CVD death and heart failure hospitalization.^19,20^ The AIHW total health price index was then used to update costs to 2014 values.^21^ The cost of CVD death and heart failure hospitalization was AUD 3642 and AUD 7136, respectively. The conservative assumption was made that only 1 heart failure hospitalization was allocated to subjects within a transition. While this makes calculations simpler, in reality multiple hospitalizations may occur within a cycle. It was also assumed that hospitalization would only occur in 50% of CVD and non-CVD deaths, and therefore only 50% of deaths would incur in hospitalization costs. This resulted in a final unit cost used in the model for both CVD and non-CVD deaths of AUD 1821. Ford et al^18^ provided other NYHA Class specific CHF background treatment costs, which were then updated using the same AIHW price index to 2014 values. These NYHA Class specific unit costs were: NYHA Class I = AUD 151, NYHA Class II = AUD 175, NYHA Class III = AUD226, and NYHA Class IV = AUD 242. Respective unit costs were applied to the relevant years of life lived of a modeled subject and formed the chronic background costs in the model.

The Australian Pharmaceutical Benefit Scheme (PBS) provided the cost of eplerenone,^22^ which is currently reimbursed for treatment of patients with postmyocardial infarction. These costs were AUD 3.76 per day for both the 25 mg and 50 mg doses, with an annual cost of AUD 1374. The Australian Medical Benefit Schedule (MBS)^23^ was used to provide the cost of tests used to monitor electrolyte and urea levels (MBS item number 66512). Two tests were conducted within the first 3 months of the model at a cost of AUD 35.60. Four tests were then used for yearly monitoring of electrolyte and urea levels at a cost of AUD 71.20.^23^

Spironolactone costs were also derived from the PBS.^24^ The cost of spironolactone per day was AUD 0.12, with an annual cost of AUD 45.29. The same ancillary costs associated with monitoring for urea and electrolytes (item number 66512) used in the eplerenone group were also attributed to the spironolactone group (AUD 71.20).^23^

### Discounting

A yearly rate of 5.0% after the first cycle^25^ was used in base-case analysis to discount years of life, costs, and QALYs. In scenario analyses we have considered a rate of 3.0% after the first cycle to discount costs and effects.

### Software

Microsoft Excel (Microsoft Corporation, Redmond, WA) and @risk (Palisade Corporation, Ithaca, NY) were used to create and run the economic model. TreeAge Pro 2014 (Triage software Inc, Williamstown, MA) was used to develop schematics and tree diagrams.

### Sensitivity Analyses

A number of deterministic sensitivity analysis (DSA), scenario, and probabilistic sensitivity analyses (PSA) were undertaken. Sensitivity analyses were undertaken with variation to key data inputs. The values of these key input parameters underlying uncertainty were deterministically varied, and this variation was captured using ±50.0% of the actual base-case value, or 95% confidence intervals, when available.

A PSA was also performed assigning probability distributions to input parameters (reflecting the ranges of variations used in the deterministic sensitivity analyses), using a Monte Carlo simulation with 10,000 iterations. Variables that were included in the Monte Carlo simulation^26,27^ were utilities (using beta distributions), costs (using uniform distributions), and transition probabilities (using triangular distributions). Costs of treatment and costs associated with monitoring of eplerenone were considered to have fixed values. Information about input variables and their uncertainty distributions are summarized in Table 2. A set of scenario analyses was considered due to the uncertainty around input parameters. A list of scenario analyses undertaken is presented in Table 3.

**TABLE 2:**
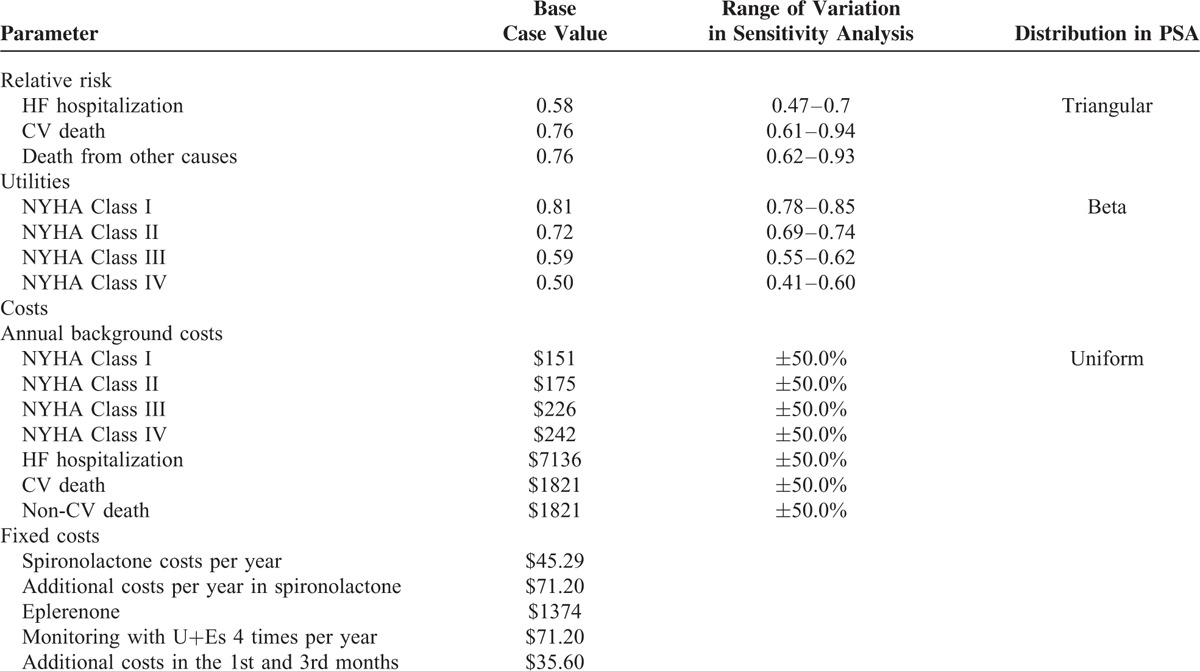
Parameter Estimates Used in the Model, Base-Case Value, Range of Variation, and Choice of Distribution

**TABLE 3:**
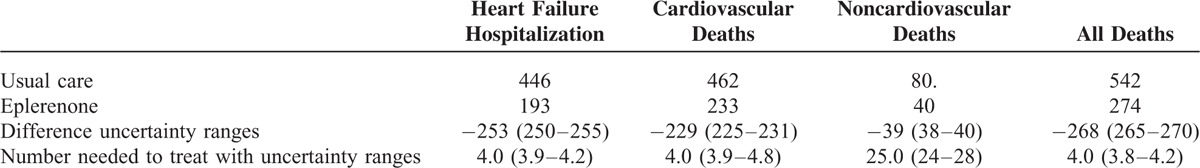
Number of Events Over 10 Years of Follow-Up Usual Care Group Versus Eplerenone

## RESULTS

Table 3 reports events per 1000 patients, and the number needed to treat (NNT). There were 263 and 291 less hospitalization and deaths respectively in the EG compared with the UCG, during 10 years of follow-up. This resulted in a NNT of 4.0 and 3.7 for hospitalization and all deaths respectively (Table 3).

The number of years of life lived and QALYs gained per person in the UCG were 6.1 and 4.4 respectively, compared with 6.3 and 4.6 respectively in the EG. Total costs per person for the UCG and the EG were AUD 4869 and AUD 11,849. The corresponding difference between the UCG and EG in terms of years lived, QALYs gained, and net costs were 0.25, 0.19, and AUD 6980, respectively (Table 4).

**TABLE 4:**
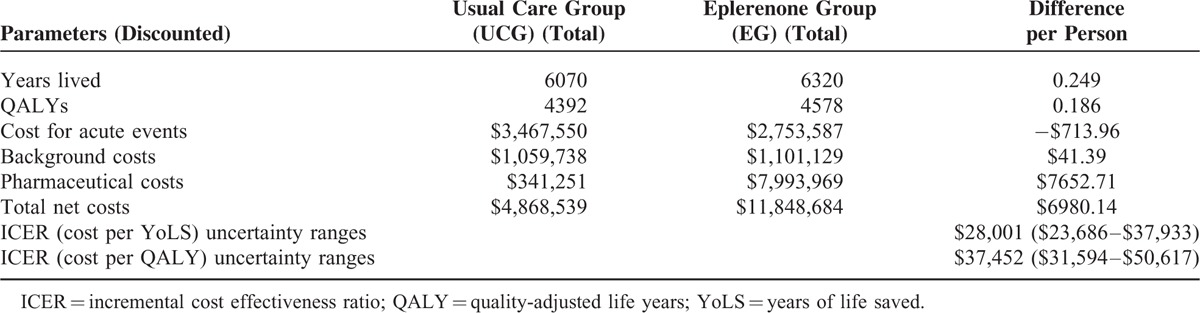
Base-Case Analysis With 95% Confidence Interval Uncertainty Ranges

The incremental cost-effectiveness ratio of eplerenone compared with the UCG was AUD 28,001 per YoLS, and AUD 37,452 per QALY gained (Table 4).

A number of DSA were performed. When the upper limits of HRs with regards to cardiovascular death were used, the ICER was above the commonly accepted threshold of AUD 50,000 per YoLS and QALY gained. In the scenario analyses, when an HR of 1.0 was considered from year 2 onward, eplerenone was found to be no longer a cost-effective strategy. All other variations in key input variables, in terms of costs, utilities, discounting and efficacy values (which are shown in Tables 5 and 6) kept the ICER below the accepted threshold of AUD 50,000 per YoLS and QALY gained.

**TABLE 5:**
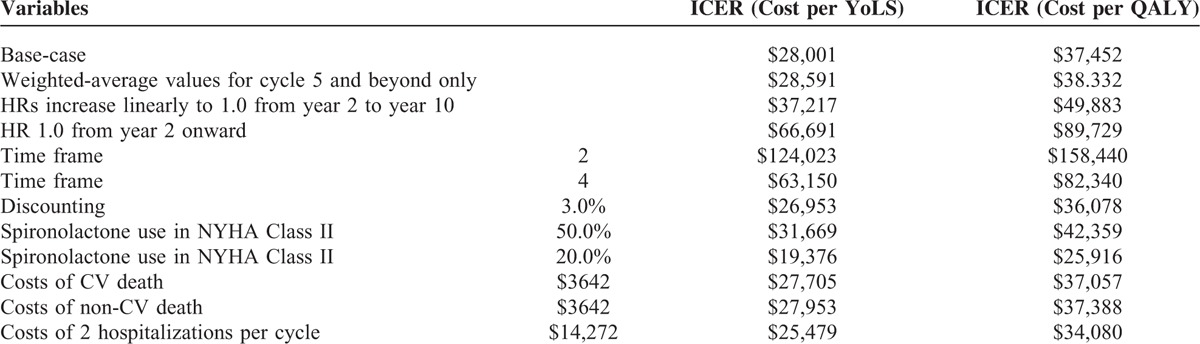
Scenario Analyses Displaying Effect of Input Variables on the ICER (Cost per YoLS and Cost per QALY)

**TABLE 6:**
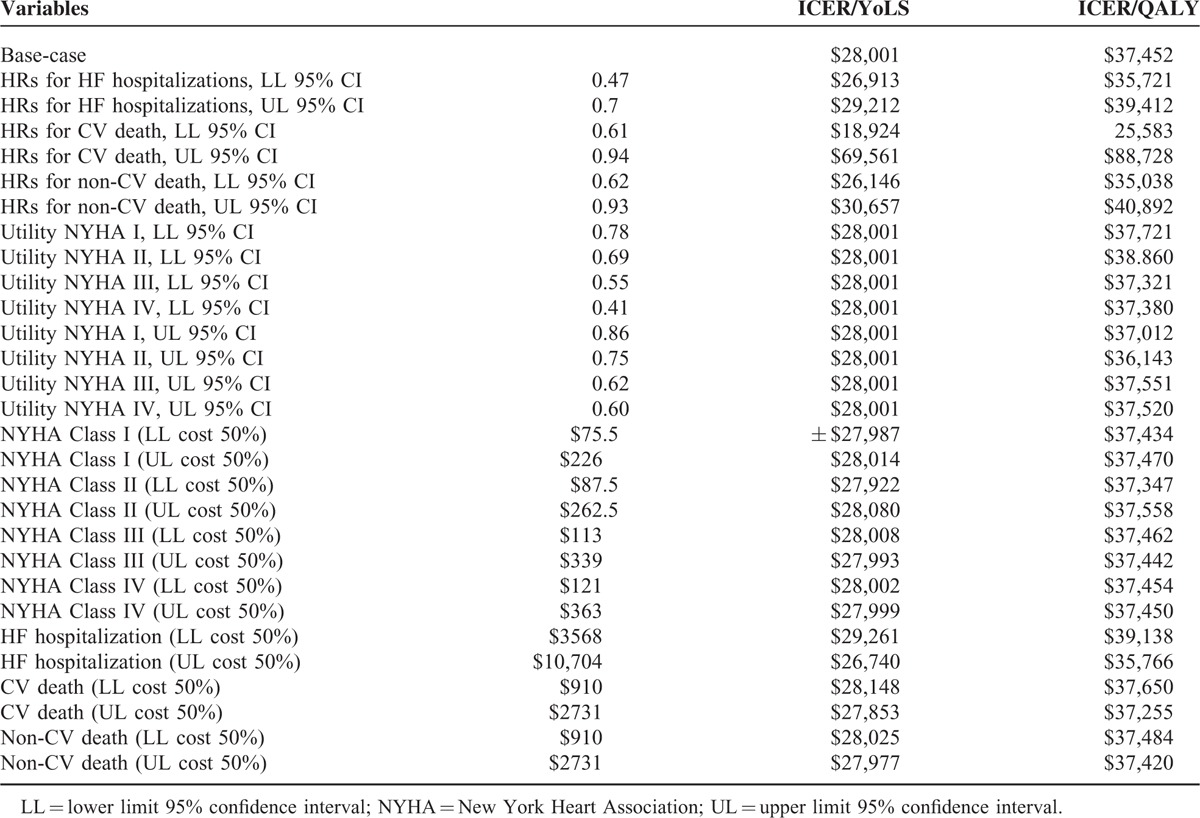
Deterministic Sensitivity Analysis (DSA) Displaying Effect of Input Variables on the ICER per YoLS and QALY

**FIGURE 2:**
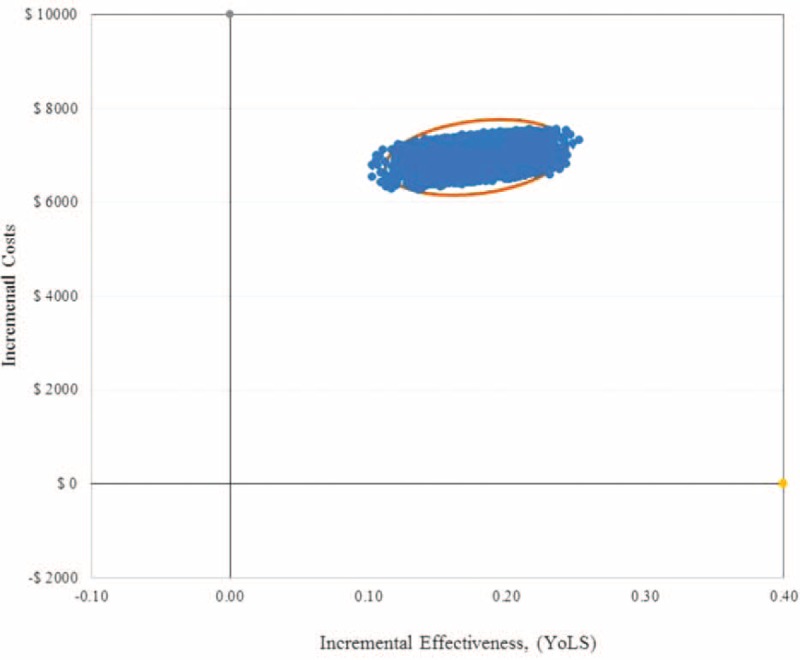
Scatter plot of incremental costs per person and incremental effectiveness. Derived from 10,000 iterations of the Monte Carlo simulation (incremental effectiveness (years of life saved (YoLS)) on the x-axis and incremental cost on the y-axis). From an Australian healthcare system perspective.

**FIGURE 3:**
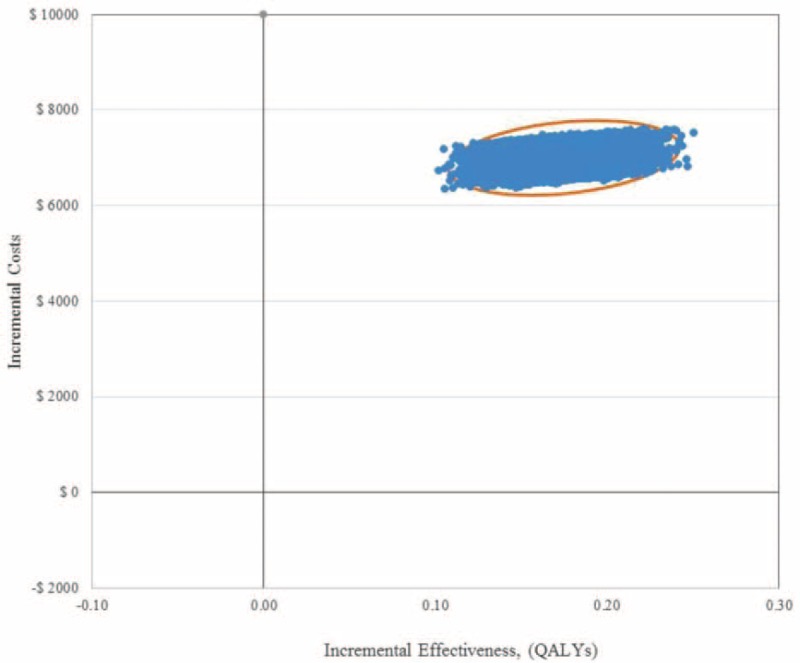
Scatter plot of incremental costs per person and incremental effectiveness. Derived from 10,000 iterations of the Monte Carlo simulation (Incremental effectiveness (quality adjusted life years (QALY) gained) on the x-axis and incremental cost on the y-axis). From an Australian healthcare system perspective.

Results of the PSA using a Monte Carlo simulation showed that eplerenone in comparison to the UCG was cost-effective in 99.0% of 10,000 iterations, when a threshold of AUD 50,000 per QALY gained was applied (Figures 2 and 3). Table 4 presents the 95% confidence interval ranges of incremental cost-effectiveness ratio, which were AUD 23,686 to $37,933 per YoLS and AUD 31,594 to AUD 50,617 per QALY gained. Scatter plots of incremental effects against incremental costs suggested that results of the modeled economic evaluation, in terms of YoLS (Figure 2) and QALY gained (Figure 3), were robust with some uncertainty around the effect parameters.

## DISCUSSION

The analyses showed that eplerenone treatment may be a cost-effective option when compared with usual care, among Australian patients with NYHA Class II CHF, with an ICER of AUD 37,452 per QALY gained. The benefits of eplerenone are attributed mostly to a decrease in the number of hospitalizations. PSA confirmed that the ICER was within accepted ranges of willingness to pay threshold as reported previously.^28–30^

No other published cost-effectiveness study to our knowledge has compared eplerenone to usual care (incorporating spironolactone) within NYHA Class II CHF treatment. However, there are 2 known cost-effectiveness analyses of eplerenone in comparison to “no active treatment” among patients initially with NYHA Class II CHF. A study by Lee et al^31^ used results from the EMPHASIS-HF trial to develop a discrete event simulation model and estimated the lifetime costs and effects of eplerenone versus standard care (no active treatment) among patients with chronic systolic HF and mild symptoms. This study^31^ was undertaken from the UK and Spanish healthcare perspective over a lifetime period, and found that the ICER was £3520 for the UK and €5532 for Spain.

The other study was undertaken by our research group, where we modeled the cost-effectiveness analysis of eplerenone compared with placebo among patients initially with NYHA Class II CHF, based on the perspective of the Australian healthcare system, with an ICER of AUD16,700 per QALY gained.^9^ As mentioned previously, this analysis did not consider the progression of patients from NYHA Class II symptoms to Class III and IV symptoms, and did not incorporate spironolactone among model subjects. These 2 assumptions are overly simplistic because in current practice, even some patients with NYHA Class II symptoms would be taking spironolactone, and the percentage of patents taking spironolactone would increase as they progressed to more severe symptom stages.

Most other studies other than Ademi et al^9^ and Lee et al^31^ looked at the use of eplerenone for patients (post-MI) with NYHA Class III and IV symptoms,^32–38^ where eplerenone was shown to be cost effective when compared with placebo,^36,37^ or not superior to Spironolactone.^38^

The major strength of our study is that it allowed for movement between NYHA Classes. Many of the assumptions made within this study were conservative, an example shown by assuming spironolactone had the same efficacy and utility as eplerenone. In reality, spironolactone may be less efficacious and may have a lower utility due to the higher instance of impotence, gynecomastia, and menstrual irregularities when compared with eplerenone.^39^

### Limitations

In terms of limitations, the majority arose due to lack of data. The lack of utility values in EMPHASIS that is discriminated by NYHA Class meant that values from the CARE-HF trial^16^ had to be used for all utility values. While this is a conservative assumption, it would be more accurate to have utility data split into NYHA Classes for placebo, spironolactone, and eplerenone treatments. The use of the EMPHASIS trial data, while comparable to the Australian population, is not a majority Australian populated source of data. This may cause minor differences in results, although it is unlikely.

In the base-case analysis, weighted average values from the EMPHASIS trial for the UCG using placebo were calculated and used for the each cycle in the 10-year time horizon of this model. However, in the scenario analyses we have used the yearly transition probabilities taken from the EMPHASIS trial data for the first 4 cycles, followed by weighted average values for cycle 5 and beyond. The corresponding ICERs in the scenario analyses were similar to the base-case analysis, which were AUD 38,332 and AUD 37,452 per QALY gained respectively.

Another limitation might be that the treatment effect beyond the duration of a clinical trial (median follow-up of 21 months in the EMPHASIS trial) is standard practice in health economic evaluations.^11,12^ In the scenario analyses, when a time frame of 2 and 4 years was applied, the corresponding ICERs were AUD 158,440 and AUD 82,340 per QALY gained respectively.

The assumption that heart failure hospitalization was only costed to occur once per transition was made to simplify calculations; however, in practise multiple hospitalizations could occur within a yearly period. In the scenario analyses, 2 hospitalizations per cycle were incorporated, and the strategy became more cost-effective with an ICER of AUD 25,479 and AUD 34,080 per YoLS and QALY gained, respectively.

The assumption that 50% of deaths would occur in hospitals was another assumption that was made due to a lack of data. To test this assumption, running the model with 100% of cardiovascular deaths occurring in hospitals gave an ICER similar to the base case results.

The results of this study can be applied to other countries with similar healthcare reimbursement systems, when costs are converted into the relevant currency and the proportion of spironolactone used in usual care is adjusted to represent clinical practice within the respective countries. As a result, this study shows that extending the use of these drugs to such patients may potentially be a cost-effective approach.

## CONCLUSIONS

In conclusion, based on our findings the addition of eplerenone to clinical treatment of CHF patients with NYHA Class II symptoms in an Australian setting may be a cost-effective approach, compared with usual care treatment.

## Supplementary Material

Supplemental Digital Content

## References

[1] Roger VL Epidemiology of heart failure. *Circ Res* 2013; 113:646–659. 23989710 10.1161/CIRCRESAHA.113.300268 PMC3806290

[2] Tendera M Epidemiology, treatment, and guidelines for the treatment of heart failure in Europe. *Eur Heart J Suppl* 2005; 7:J5–J9.

[3] Krum H Abraham WT Heart failure. *Lancet* 2009; 373:941–955. 19286093 10.1016/S0140-6736(09)60236-1

[4] Krum H Driscoll A Management of heart failure. *Med J Aust* 2013; 199:334–339. 23992190 10.5694/mja12.10993

[5] National Heart Foundation of Australia and the Cardiac Society of Australia and New Zealand (Chronic Heart Failure Guidelines Expert Writing Panel). Guidelines for the prevention, detection and management of chronic heart failure in Australia. Updated October 2011.

[6] Stewart S Hart CL Hole DJ Population prevalence, incidence, and predictors of atrial fibrillation in the Renfrew/Paisley study. *Heart* 2001; 86:516–521. 11602543 10.1136/heart.86.5.516 PMC1729985

[7] Bui AL Horwich TB Fonarow GC Epidemiology and risk profile of heart failure. *Nat Rev Cardiol* 2011; 8:30–41. 21060326 10.1038/nrcardio.2010.165 PMC3033496

[8] Zannad F McMurray JJV Krum H Eplerenone in patients with systolic heart failure and mild symptoms. *N Engl J Med* 2011; 364:11–21. 21073363 10.1056/NEJMoa1009492

[9] Ademi Z Pasupathi K Krum H Cost effectiveness of eplerenone in patients with chronic heart failure. *Am J Cardiovasc Drugs* 2014; 14:209–216. 24610254 10.1007/s40256-014-0066-3

[10] Briggs A Sculpher M An introduction to Markov modelling for economic evaluation. *Pharmacoeconomics* 1998; 13:397–409. 10178664 10.2165/00019053-199813040-00003

[11] Caro JJ Briggs AH Siebert U Modeling good research practices—overview: a report of the ISPOR-SMDM Modeling Good Research Practices Task Force—1. *Value Health* 2012; 15:796–803. 22999128 10.1016/j.jval.2012.06.012

[12] Schwenkglenks M Toward TJ Plent S Cost-effectiveness of bivalirudin versus heparin plus glycoprotein IIb/IIIa inhibitor in the treatment of acute ST-segment elevation myocardial infarction. *Heart* 2012; 98:544–551. 22313548 10.1136/heartjnl-2011-301323

[13] Briggs A Claxton K Sculpher M Decision Modelling for Health Economic Evaluation. Oxford, UK: Oxford University Press; 2007.

[14] Parnicka A Wizner B Fedyk-Lukasik M Knowledge about heart failure in primary care: need for strengthening of continuing medical education. *Cardiol J* 2013; 20:356–363. 23913453 10.5603/CJ.2013.0093

[15] Australian Institute of Health and Welfare. *National GRIM Books*. Available at: http://www.aihw.gov.au/national-grim-books/ Accessed May 2, 2013.

[16] Cleland JG Daubert JC Erdmann E The effect of cardiac resynchronization on morbidity and mortality in heart failure. *N Engl J Med* 2005; 352:1539–1549. 15753115 10.1056/NEJMoa050496

[17] Yao G Freemantle N Calvert MJ The long-term cost-effectiveness of cardiac resynchronization therapy with or without an implantable cardioverter-defibrillator. *Eur Heart J* 2007; 28:42–51. 17110403 10.1093/eurheartj/ehl382

[18] Ford E Adams J Graves N Development of an economic model to assess the cost-effectiveness of hawthorn extract as an adjunct treatment for heart failure in Australia. *BMJ Open* 2012; 2: 10.1136/bmjopen-2012-001094 PMC3437431 22942231

[19] Department of Health and Ageing. AR-DRG version 6.0. Canberra: Australian government; 2012 [cited August 31, 2012]. Available at: http://www.health.gov.au/internet/main/publishing.nsf/Content/AR-DRG-Version_6.0.

[20] Data collections. Public Sector Estimated Cost Weights Round 11 AR-DRG v5.1 (Excel 277 KB). Available at: http://www.healthemergency.gov.au/internet/main/publishing.nsf/Content/Round_11-cost-reports Accessed April 18, 2013.

[21] Australian Institute of Health and Welfare. Health Expenditures Australia 2011–2012. AIHW. Available at: https://http://www.aihw.gov.au/WorkArea/DownloadAsset.aspx?id=60129544656 Accessed February 1, 2014.

[22] Pharmaceutical Benefit Scheme (PBS). Available at: http://www.pbs.gov.au/pbs/home Accessed July 7, 2013.

[23] Medicare Australia. Available at: http://www.mbsonline.gov.au/ Accessed December 11, 2013.

[24] Pharmaceutical Benefit Scheme (PBS). Available at: http://www.pbs.gov.au/medicine/item/2339D Accessed April 12, 2014.

[25] Severens JL Milne RJ Discounting health outcomes in economic evaluation: the ongoing debate. *Value Health* 2004; 7:397–401. 15449631 10.1111/j.1524-4733.2004.74002.x

[26] Briggs A Sculpher M Buxton M Uncertainty in the economic evaluation of health care technologies: the role of sensitivity analysis. *Health Econ* 1994; 3:95–104. 8044216 10.1002/hec.4730030206

[27] O’Hagan A McCabe C Akehurst R Incorporation of uncertainty in health economic modelling studies. *Pharmacoeconomics* 2005; 23:529–536. 15960550 10.2165/00019053-200523060-00001

[28] Raftery J Review of NICE's recommendations, 1999-2005. *BMJ* 2006; 332:1266–1268. 16735341 10.1136/bmj.332.7552.1266 PMC1471962

[29] Kirchmann K Kirchmann M Ademi Z Predictors of successfully listing on the PBS: a 2012 update. Poster presentation. ISPOR 5th Asia-Pacific Conference. 2012. Taipei, Taiwan.

[30] Simoens S Health economic assessment cost-effectiveness thresholds and other decision criteria. *Int J Environ Res Public Health* 2010; 7:1835–1840. 20617062 10.3390/ijerph7041835 PMC2872336

[31] Lee D Wilson K Akehurst R Cost-effectiveness of eplerenone in patients with systolic heart failure and mild symptoms. *Heart* 2014; 100:1681–1687. 24993605 10.1136/heartjnl-2014-305673 PMC4215293

[32] Zhang Z Mahoney EM Kolm P Cost effectiveness of eplerenone in patients with heart failure after acute myocardial infarction who were taking both ACE inhibitors and beta-blockers: subanalysis of the EPHESUS. *Am J Cardiovasc Drugs* 2010; 10:55–63. 20104935 10.2165/11319940-000000000-00000

[33] Croom KF Plosker GL Eplerenone: a pharmacoeconomic review of its use in patients with post-myocardial infarction heart failure. *Pharmacoeconomics* 2005; 23:1057–1072. 16235978 10.2165/00019053-200523100-00008

[34] de Pouvourville G Solesse A Beillat M Cost-effectiveness analysis of aldosterone blockade with eplerenone in patients with heart failure after acute myocardial infarction in the French context: the EPHESUS study. *Arch Cardiovasc Dis* 2008; 101:515–521. 19041835 10.1016/j.acvd.2008.09.001

[35] McKenna C Walker S Lorgelly P Cost-effectiveness of aldosterone antagonists for the treatment of post-myocardial infarction heart failure. *Value Health* 2012; 15:420–428. 22583451 10.1016/j.jval.2012.01.004

[36] Szucs TD Holm MV Schwenkglenks M Cost-effectiveness of eplerenone in patients with left ventricular dysfunction after myocardial infarction—an analysis of the EPHESUS study from a Swiss perspective. *Cardiovasc Drugs Ther* 2006; 20:193–204. 16775667 10.1007/s10557-006-8282-y

[37] Weintraub WS Zhang Z Mahoney EM Cost-effectiveness of eplerenone compared with placebo in patients with myocardial infarction complicated by left ventricular dysfunction and heart failure. *Circulation* 2005; 111:1106–1113. 15723981 10.1161/01.CIR.0000157146.86758.BC

[38] Chatterjee S Moeller C Shah N Eplerenone is not superior to older and less expensive aldosterone antagonists. *Am J Med* 2012; 125:817–825. 22840667 10.1016/j.amjmed.2011.12.018

[39] Pitt B Williams G Remme W The EPHESUS trial: eplerenone in patients with heart failure due to systolic dysfunction complicating acute myocardial infarction. Eplerenone Post-AMI Heart Failure Efficacy and Survival Study. *Cardiovasc Drugs Ther* 2001; 15:79–87. 11504167 10.1023/a:1011119003788

