# Supplementary material for: Cost-Effectiveness of Eplerenone Compared to Usual Care in Patients With Chronic Heart Failure and NYHA Class II Symptoms, an Australian Perspective
Source: Medicine (Baltimore). 2016 May 6;95(18):e3531. doi: 10.1097/MD.0000000000003531 (PMC4863773; doi:10.1097/MD.0000000000003531)

Appendix 1. Characteristics of participants in the EMPHASIS trial

| Characteristic | Eplerenone Group  (N=1364) | Placebo Group  (N=1373) |
| --- | --- | --- |
| Age - years, mean (SD) | 68.7 (7.7) | 68.6 (7.6) |
| Females | 22.7% | 21.9% |
| Race  White  Black  Asian  Other | 82.6%  2.7%  11.6%  3.1% | 83.1%  2.2%  11.5%  3.2% |
| Heart rate - bpm, mean (SD) | 72 (12) | 72 (13) |
| Blood pressure - mmHg, mean | 124/75 | 124/75 |
| LVEF - mean (SD) | 26.2% (4.6) | 26.1% (4.7) |
| Previous HF hospitalisation | 52.3% | 52.9% |
| Previous myocardial infarction | 50.3% | 50.6% |
| Previous stroke | 10.0% | 9.2% |
| Diabetes | 33.7% | 29.1% |
| Atrial fibrillation or flutter | 30.0% | 31.7% |
| Medications  ACE inhibitor  ARB  Beta-blocker  Diuretic  Digoxin | 78.3%  19.1%  86.6%  84.3%  26.6% | 76.8%  19.4%  86.9%  85.7%  27.5% |

Appendix 2. Probabilities of death among Australians in 2007, by five-year age-groups


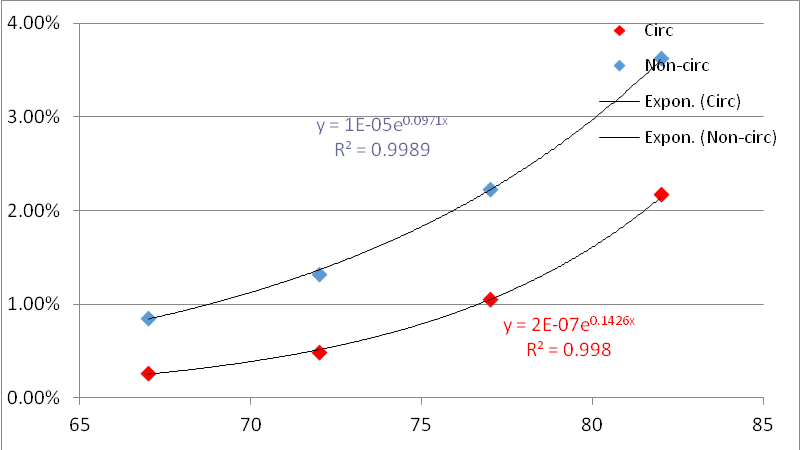

Supplement: Supplemental Digital Content [file medi-95-e3531-s001.doc]
